# Interleukin-4 Supports the Suppressive Immune Responses Elicited by Regulatory T Cells

**DOI:** 10.3389/fimmu.2017.01508

**Published:** 2017-11-14

**Authors:** Wei-Cheng Yang, Yih-Shiou Hwang, Ying-Yu Chen, Chao-Lin Liu, Chia-Ning Shen, Wei-Hsin Hong, Sheng-Min Lo, Chia-Rui Shen

**Affiliations:** ^1^Department and Graduate Institute of Medical Biotechnology and Laboratory Science, College of Medicine, Chang Gung University, Taoyuan City, Taiwan; ^2^Graduate Institute of Biomedical Sciences, College of Medicine, Chang Gung University, Taoyuan City, Taiwan; ^3^Department of Medicine, College of Medicine, Chang Gung University, Taoyuan City, Taiwan; ^4^Department of Ophthalmology, Lin-Kou Chang Gung Memorial Hospital, Taoyuan City, Taiwan; ^5^Department of Chemical Engineering, Ming Chi University of Technology, New Taipei City, Taiwan; ^6^College of Engineering, Chang Gung University, Taoyuan City, Taiwan; ^7^Genomics Research Center, Academia Sinica, Taipei, Taiwan

**Keywords:** regulatory T cell, interleukin-4, granzyme, immunosuppression, cell survival

## Abstract

Interleukin-4 (IL-4) has been considered as one of the tolerogenic cytokines in many autoimmune animal models and clinical settings. Despite its role in antagonizing pathogenic Th1 responses, little is known about whether IL-4 possesses functions that affect regulatory T cells (Tregs). Tregs are specialized cells responsible for the maintenance of peripheral tolerance through their immune modulatory capabilities. Interestingly, it has been suggested that IL-4 supplement at a high concentration protects responder T cells (Tresps) from Treg-mediated immune suppression. In addition, such supplement also impedes TGF-β-induced Treg differentiation *in vitro*. However, these phenomena may contradict the tolerogenic role of IL-4, and the effects of IL-4 on Tregs are therefore needed to be further elucidated. In this study, we utilized IL-4 knockout (KO) mice to validate the role of IL-4 on Treg-mediated immune suppression. Although IL-4 KO and control animals harbor similar frequencies of Tregs, Tregs from IL-4 KO mice weakly suppressed autologous Tresp activation. In addition, IL-4 deprivation impaired the ability of Tregs to modulate immune response, whereas IL-4 supplementation reinforced IL-4 KO Tregs in their function in suppressing Tresps. Finally, the presence of IL-4 was associated with increased cell survival and granzyme expression of Tregs. These results suggest the essential role of IL-4 in supporting Treg-mediated immune suppression, which may benefit the development of therapeutic strategies for autoimmune diseases.

## Introduction

The effector and suppressor immune responses appear to be maintained in a well-tuned and active equilibrium, leading to immune system homeostasis. Imbalance between Th1 and Th2 responses results in various diseases, including autoimmune diseases, infection, and allergy (1). Th1 effectors can be both initiators and effectors in autoimmune diseases (2–5). Various therapeutic strategies that target reinstatement of the Th1 and Th2 balance have been developed and proven effective for ameliorating autoimmune disorders *via* different mechanisms.

Interleukin-4 (IL-4), which is mainly secreted by activated T cells, is the dominant Th2 deviator for initiating and expanding humoral immunity. Despite its role in promoting B cell maturation and survival, the antagonistic nature of IL-4 on Th1 polarization makes it a potential therapeutic agent. Studies performed in murine models have indicated that autoimmune-related inflammation can be controlled by IL-4 treatment through its pleiotropic effects on the immune system, which include inducing alternative activated macrophages (6, 7), equipping macrophages with regulatory functions (8), and combining with retinoic acid to endow dendritic cells with immune modulatory properties (9). Increment of IL-4 has been demonstrated in autoimmune animal models with ameliorated disease (10). In fact, the administration of IL-4 has relieved psoriasis progression by inducing Th2 responses in patients with persistent disease (11). Suppression of the Th1 response through nasal administration of autoantigen-derived peptides has been proven to protect against disease development in spontaneous autoimmune disease model (10, 12, 13). Moreover, the treatments promote the formation of a tolerogenic CD4 T cell population (12, 13), which at least partially contributed to the therapeutic effect. In addition, the lack of IL-4 impedes the generation of graft tolerance in a murine transplantation model (14). Together, these findings indicate that IL-4 is beneficial for the maintenance and establishment of peripheral tolerance.

CD4^+^CD25^+^Foxp3^+^ regulatory T cells (Tregs) play important roles in maintaining peripheral tolerance with their immune suppressive capabilities (15, 16), while decreased numbers or disruption of the functionalities of Tregs may lead to severe autoimmune diseases in both mice and humans (17–22). Although little or no effect of IL-4 on CD4^+^CD25^+^ Tregs in suppressing polyclonal T cell activation was reported (23), other studies have suggested several positive roles of IL-4 on Tregs or Treg-mediated immune suppression (24–28). For example, preincubation of natural Tregs with IL-4 enhanced their suppressive capabilities (27), perhaps through the preservation of their Foxp3 expression after anti-CD3-mediated cell activation (24). Also, with IL-4 supplement, *ex vivo* alloantigen-activated Tregs turned into potent alloantigen suppressors against rejection (28). Alternatively, IL-4 treatment was shown to elicit nTreg proliferation and to prevent their apoptosis (24, 25, 27). In a graft-versus-host disease animal model, donor Tregs were proliferated *in vivo* by an IL-4 dependent mechanism harbored by host natural killer T cells thereby preventing the host from the disease (29). Because the intact STAT6, a downstream signal transducer of IL-4 receptor, signaling pathway is required for the development of human inducible Tregs (iTregs) (30) and in agonist-driven murine Treg differentiation (31), IL-4 appears to correlate with the presence of Tregs. By contrast, IL-4 dampened the differentiation process of iTregs by downregulating their Foxp3 expression (32–34), and the deficiency of STAT6 consequently enriched the Treg population in a murine airway inflammation model (35). Also, with IL-4 treatment, the effector T cells became insensitive to Treg-mediated suppression by activating the IL-4 dependent signaling pathway along with upregulating antiapoptotic Bcl-2 (25, 36, 37).

Given that both positive and negative effects of IL-4 on the maintenance of immune homeostasis have been reported, the role of IL-4 on Treg-mediated immune suppression needs to be further elucidated. The aim of this study is to identify the beneficial role of IL-4 on Treg-mediated immune suppression. Here, we demonstrate the requirement of IL-4 for optimal regulation of T cell proliferation by harboring Tregs and responder T cells (Tresps) from WT and IL-4 knockout (KO) mice for the suppression assay. In addition, both removal and administration of IL-4 affect the suppressive effects elicited by Tregs, and the positive effects of IL-4 on the immune suppression may be shown through the maintenance of cell survival and enhancement of granzyme expression in Tregs.

## Results

### Deficiency of IL-4 Affects the Suppressive Immune Responses Elicited by Tregs

**Figure 1:**
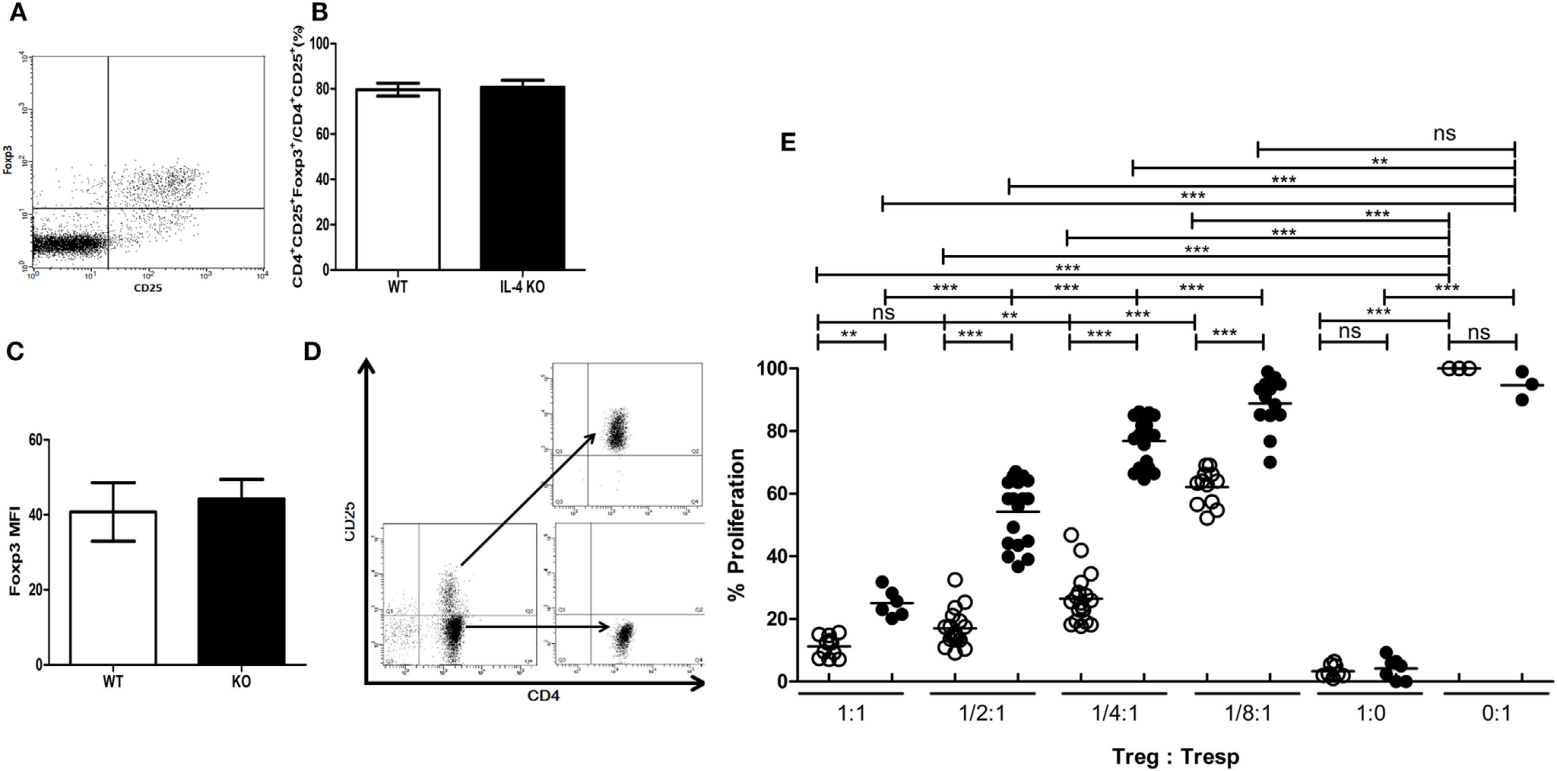
Incompetence in exerting *in vitro* immune suppression of interleukin-4 (IL-4)-deficient regulatory T cells (Tregs). **(A)** A representative dot plot demonstrating the strategy for analyzing the presence of CD25^+^Foxp3^+^ Tregs in the splenic T cell population (gate on CD4^+^ T cells). **(B)** The percentages of Tregs (Foxp3^+^) among CD4^+^CD25^+^ T cells and **(C)** the intensity of Foxp3 expression in CD4^+^CD25^+^Foxp3^+^ T cells in healthy IL-4 knockout (KO) (*n* = 6) and WT (*n* = 6) mice were analyzed by flow cytometry. Results are given as the mean ± SEM. **(D)** Tregs and responder T cells (Tresps) were sorted from CD4^+^ T cells from IL-4 KO and WT mice for the *in vitro* suppression assay. **(E)** The suppressive capability of IL-4 KO and WT Tregs was compared in suppressing their autologous Tresps. Data are representative of three independent experiments. The proliferation of WT Tresps alone was used as the reference for cell proliferation. Results are presented as the relative percentage of cell proliferation to corresponding Tresps alone. ○, WT Treg:WT Tresp; ●, IL-4 KO Treg:IL-4 KO Tresp (*p < 0.05; **p < 0.01; and ***p < 0.001).

To understand if deficiency of IL-4 affects the suppressive immune responses elicited by Tregs, we first investigated whether systemic IL-4 deficiency affects Treg development. First, the composition of CD4^+^CD25^+^Foxp3^+^ Tregs in healthy IL-4 KO and WT mice was similar (Figures 1A,B). Second, Foxp3 expression in Tregs was similar between cells from WT and IL-4 KO mice (Figure 1C), indicating the IL-4 deficiency did not disrupt the Treg pool in the steady state. We further tested Tregs from healthy WT and IL-4 KO mice for their capacity to suppress autologous Tresps. Tregs and Tresps were prepared by negative selection *via* magnetic enrichment for the CD4^+^ population and were then sorted to separate CD25^−^ and CD25^+^ cells by FACSAria (Figure 1D). Surprisingly, IL-4 KO Tregs demonstrated inferior suppressive capabilities when suppressing autologous Tresps *in vitro* (Figure 1E), in contrast to WT Tregs. In particular, WT Tregs maintained similar suppression on Tresp proliferation with Treg:Tresp ratios of 1:1 and 1:2, whereas the suppressive capacity of Tregs from IL-4 KO mice was significantly reduced from a Treg:Tresp ratio of 1:2. Moreover, it is evident that the proliferation rates of Tresps from IL-4 KO and WT mice were 77 versus 26%, respectively, when the Treg:Tresp ratio was 1:4, indicating that Tregs from IL-4 KO mice had much worse suppression than those from WT mice.

### IL-4 KO Tregs Demonstrate an Inferior Phenotype in Mediating Immune Suppression *In Vitro*

**Figure 2:**
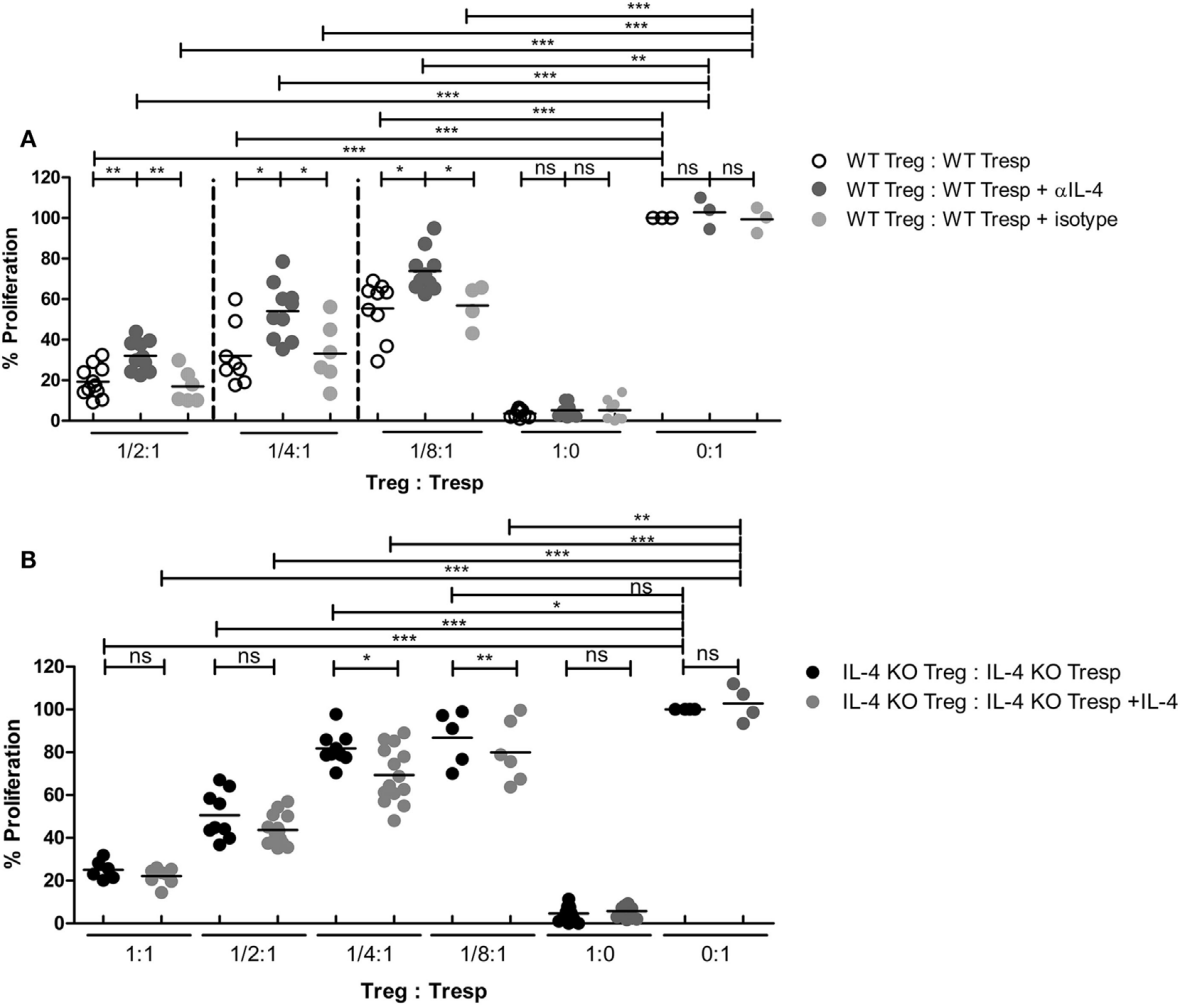
Loss of interleukin-4 (IL-4) deteriorated the immune suppressive responses elicited by regulatory T cells (Tregs). Tregs and responder T cells (Tresps) were sorted from IL-4 knockout (KO) or WT mice, as described in Figure 1D, for the suppression assay. **(A)** The effect of IL-4 removal on the suppressive capability of WT Tregs. **(B)** The effect of IL-4 supplement on the suppressive capability of IL-4 KO Tregs. Data are representative of three **(A)** and four **(B)** independent experiments. The proliferation of WT Tresps alone **(A)** and IL-4 KO Tresps alone **(B)** were used as the reference for cell proliferation. Results are presented as the relative percentage of cell proliferation to corresponding Tresps alone (*p < 0.05; **p < 0.01; and ***p < 0.001).

**Figure 3:**
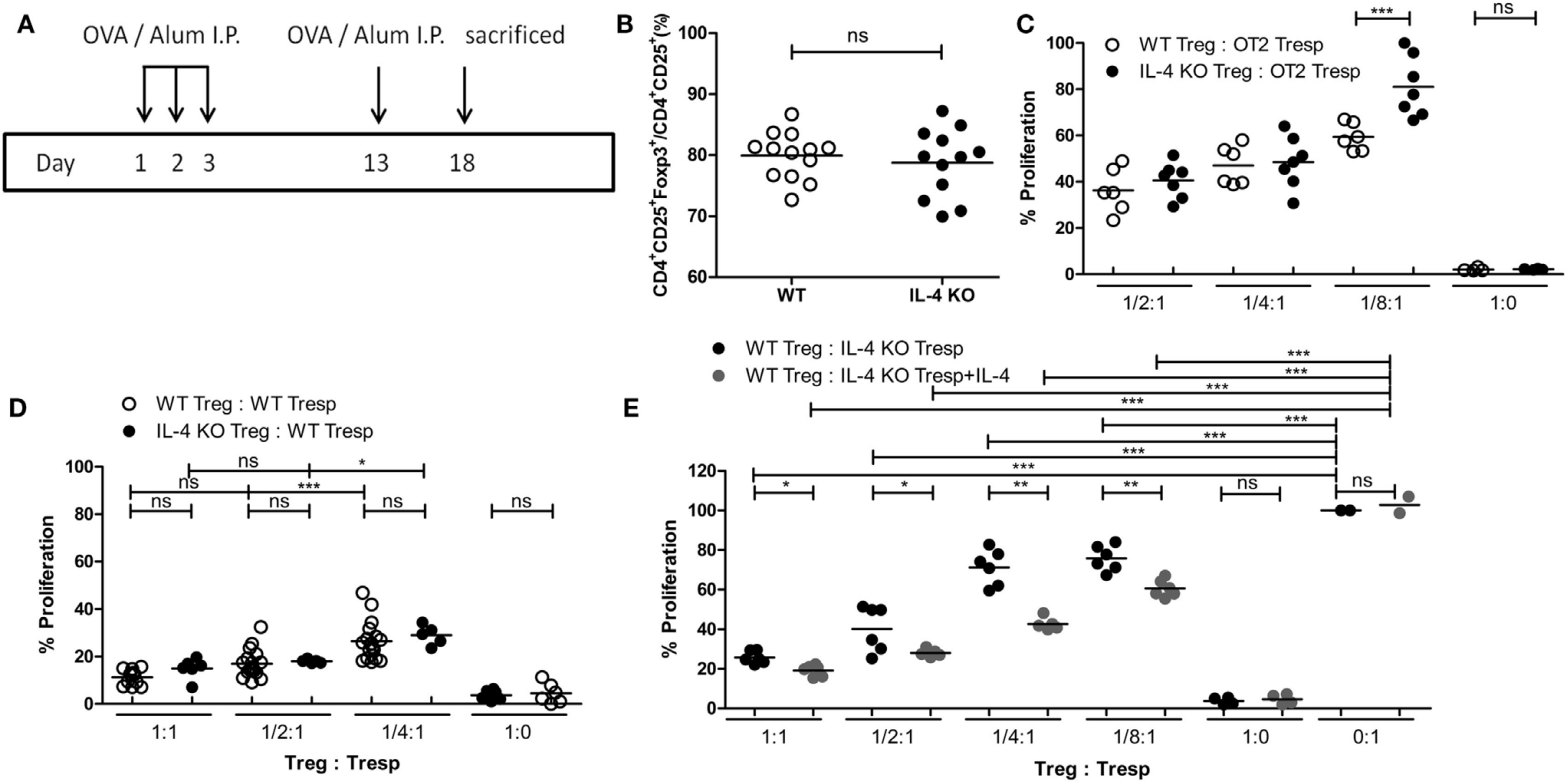
Insufficiency in mediating *in vitro* antigen-specific immune suppression of interleukin-4 (IL-4)-deficient regulatory T cells (Tregs). **(A)** Schematic diagram illustrating the protocol for enriching OVA-specific Tregs from WT and IL-4 knockout (KO) mice. **(B)** The percentages of Foxp3 expressing cells among CD4^+^CD25^+^ T cells in OVA-immunized WT (*n* = 13) and IL-4 KO (*n* = 12) mice were analyzed by flow cytometry. **(C)** The suppressive capability of IL-4 KO and WT Tregs in exerting antigen-specific suppression. **(D)** The suppressive capability of IL-4 KO Tregs in suppressing WT responder T cells (Tresps). **(E)** The suppressive capability of WT Tregs in suppressing IL-4 KO Tresps. Results are presented as the relative percentage of cell proliferation to OT2 Tresps alone **(C)**, WT Tresps alone **(D)**, and IL-4 KO Tresps alone **(E)**. Data are representative of two **(B,C,E)** and three **(D)** independent experiments (*p < 0.05; **p < 0.01; and ***p < 0.001).

Since IL-4 is important for Treg-mediated immune suppression *in vitro*, removal of IL-4 also deteriorates the immune suppressive capabilities of WT Tregs. Indeed, Tregs from WT mice gradually lost their suppressive capabilities when IL-4 neutralizing antibodies were introduced (Figure 2A). In addition, we found that re-introduction of recombinant IL-4 at 40 pg/mL, which is determined by referring to the amount of IL-4 secreted by WT Tresps after anti-CD3 and anti-CD28 mediated stimulation (Table 1), could enhance the suppressive ability of IL-4 KO Tregs for *in vitro* suppression assays (Figure 2B), indicating that the presence of IL-4 could be crucial for Tregs to perform their regulatory functions. Next, the question may be raised about whether the immune suppressive defect of IL-4 KO Tregs was limited to an antigen non-specific manner. To clarify whether IL-4 has extensive effects on Treg-mediated antigen-specific immune suppression, OT2 Tresps were utilized. A recall immune response to OVA was established in IL-4 KO and WT mice (Figure 3A), and their Tregs were harvested and enriched in the end of the immunizing protocol depending on the expression of CD4 and CD25. The percentages of Foxp3 expressing cells among CD4^+^CD25^+^ T cells in OVA sensitized mice were analyzed (Figure 3B), and the resultant Tregs were mixed with OT2 CD4^+^ T cells at different ratios. Mixed cells were activated by OT2 peptide for 72 h and then tested for cell proliferation. Tregs from IL-4 KO mice demonstrated an inferior phenotype when suppressing OT2 cells (Figure 3C), indicating that the defect in IL-4 KO Tregs existed in both antigen non-specific and antigen-specific settings. It may be argued that there seemed to be no difference in the suppressive effect of IL-4 KO and WT Tregs that were cocultured with WT Tresps in the suppression assays with CD3 activation (Figure 3D). Interestingly, WT Tregs failed to properly suppress IL-4 KO Tresps in the cross-suppression assay. However, the suppressive capabilities of WT Tregs recovered when IL-4 was supplied at 40 pg/mL (Figure 3E). It is noteworthy that we could not detect IL-4 secreted by WT Tregs or OT2 CD4^+^ T cells after stimulation (Table 1). Given that ^3^H-thymidine incorporation assay indiscriminatingly measures cell proliferation in the suppression assay, we therefore validated the proliferation of Tregs and Tresps from WT and IL-4 KO mice alone under different experimental circumstances to strengthen the results we obtained from suppression assays. We found that there were no significant differences between the proliferation of Tresps from WT and IL-4 KO mice. Moreover, though WT and IL-4 KO Tregs both slightly proliferate after TCR engagement, we cannot observe any significant difference of the proliferation of these cells. In addition, it is obvious that the proliferation of Tregs contributed little to the overall cell proliferation we detected in the suppression assay (Figure S1 in Supplementary Material).

**Table 1:** The amount of IL-4 (pg/mL) produced by cells after stimulation.

	0:1	1/2:1	1/4:1	1/8:1	
WT Treg:OT2 Tresp	ND				
IL-4 KO Treg:OT2 Tresp	ND				

	**1:0**	**0:1**	**1:1**	**1/2:1**	**1/4:1**

WT Treg:WT Tresp	ND	43.41 ± 4.80	ND	ND	5.48 ± 1.58
IL-4 KO Treg:WT Tresp	ND	43.41 ± 4.80	ND	ND	ND

*Treg, regulatory T cell; Tresp, responder T cell; KO, knockout; IL-4, interleukin-4*.

### Profound Effects of IL-4 on Tregs and Treg-Mediated Immune Suppression

**Figure 4:**
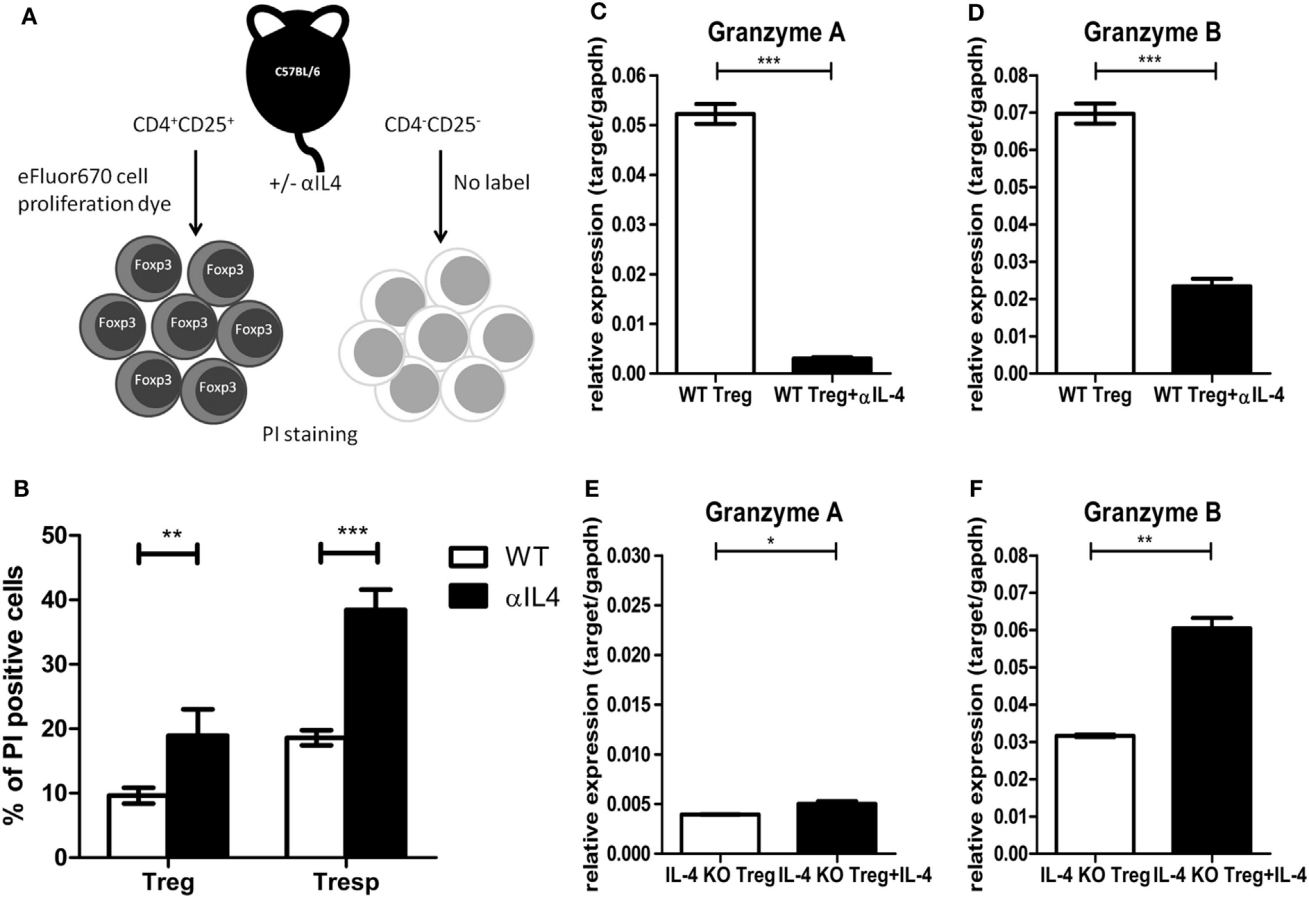
Interleukin-4 (IL-4) deficiency resulted in increased cell death and decreased granzyme expression in regulatory T cells (Tregs). **(A)** The schematic diagram shows the sorting strategy after culture by labeling Tregs with eFluor 670 cell proliferation dye. Tregs and responder T cells (Tresps) from IL-4 knockout (KO) and WT mice were prepared and cocultured 1:1 for 72 h. The IL-4 neutralization antibody or recombinant IL-4 was supplied as indicated. **(B)** The effect of anti-IL-4 on the cell death of Tregs and Tresps was determined by PI staining. **(C–F)** The differences of granzyme A and granzyme B expression levels were compared by real-time RT-PCR in Tregs from IL-4 KO or WT mice with supplementation of IL-4 neutralization antibody or recombinant IL-4. Data are representative of two independent experiments. Results are presented as the mean ± SEM (*p < 0.05, **p < 0.01; and ***p < 0.001).

The above results from *in vitro* suppression assays clearly demonstrated that IL-4 deficiency could result in inferior suppressive capabilities of Tregs. Given that IL-4, such as other γ-chain cytokines, is known for promoting Treg survival (38), we used an *in vitro* single labeling strategy to identify the impact of IL-4 deficiency on Treg survival (Figure 4A). The cell death percentages for Tregs and Tresps were determined by propidium iodide staining. The cell death percentages increased in both Tregs and Tresps that were treated with anti-IL-4 antibody during activation (Figure 4B). In addition to the increased cell death of Tregs after IL-4 neutralization, we focused on the expression of effector molecules, which may contribute to Treg-mediated immune modulation. Compared with Tregs from WT mice, significantly lower expression of granzyme B was identified in both Tregs treated with anti-IL-4 neutralization antibody and Tregs from IL-4 KO mice. Both granzyme B and granzyme A are important for Treg-mediated immune regulation of pathogenic immune responses (39–41). We validated the expression of both granzyme A and granzyme B by RT-qPCR in Tregs 72 h after they were cocultured 1:1 with Tresps. Neutralization of IL-4 resulted in significantly decreased expression of both granzyme A and granzyme B in Tregs from WT mice (Figures 4C,D), whereas the expression of granzyme A and granzyme B significantly increased in Tregs from IL-4 KO mice that were supplemented with exogenous IL-4 (Figures 4E,F). These results suggested that IL-4 contributes to the induction of granzyme A and granzyme B in Tregs and may consequently affect immune suppression.

## Discussion

Here, we demonstrated that the presence of IL-4 is important for the suppressive immune response elicited by Tregs. The inferior suppression of Tresp proliferation was obtained in Tregs not only from IL-4 KO mice but also from WT mice treated with the anti-IL-4 neutralization antibody. Also, supplementation of recombinant IL-4 appeared to strengthen the function of Tregs from IL-4 KO mice. Such a defective phenotype of IL-4-deficient Tregs might be due to the loss of granzyme A and granzyme B expression.

Although the pathological role of IL-4 and Th2 immune response in autoimmune development has been demonstrated in various autoimmune disorders, the role of IL-4 in autoimmunity remains controversial. Genetic polymorphisms of IL-4 and its signaling downstream element, STAT6, are also related to systemic lupus erythematosus (SLE) in humans (42), and increased plasma IL-4 can be detected in patients with SLE or rheumatoid arthritis (43). By contrast, IL-4 may also play regulatory roles during autoimmune recovery. In rats with experimental autoimmune encephalitis, unlike in the peak of disease progression, IL-4 can be identified in the brain tissue from rats that are naturally recovering from the disease, suggesting the regulatory role of IL-4 in disease remission (44). Also, the STAT6 deficiency in a Lyn^−/−^ background (Lyn is the negative regulator of Th2 immune response) facilitated the development of autoimmune diseases compared with those with intact STAT6 expression (45). These studies suggested that IL-4 and IL-4R signaling plays an indispensable role of in regulating disease development.

The IL-4 level seems to determine the impact of IL-4 on the immune system. In this study, we supplied IL-4 KO Tregs with a physiologically relevant amount of IL-4 (Table 1), which partially restored their defective phenotype (Figure 2B). Nonetheless, in accordance with previous reports (36, 37), IL-4 that was supplied at higher concentrations induced vigorous cell proliferation and deteriorated both WT and IL-4 KO Treg-mediated immune suppression (Figure S2 in Supplementary Material). Systemic atopic expression of IL-4 resulted in the development of autoimmune-like disorders, including autoimmune hemolytic anemia, glomerulonephritis, and Ig deposits in the kidney, which were primarily mediated by elevated levels of autoantibodies (46). Interestingly, in a murine model of lung inflammation, which is a Th2 dominant allergic response, STAT6^−/−^ mice were completely resistant to disease induction, which is partially due to the increased number of Tregs (35). These findings support the role of excessive IL-4 and STAT6 activation in inhibiting Treg-mediated immune suppression. By contrast, low expression of IL-4 under the control of IgH promoter restricted in B cells (47) completely protected the genetic autoimmune prone (NZWxC57BL/6 *Yaa*) murine model of SLE against disease progression. IL-4 expression of these B cells has been shown as sufficient to downmodulate the pathogenic Th1 immune responses in these animals (48). These findings also indicate that a low IL-4 level is sufficient to tune immune responses through various mechanisms and the IL-4 level determines the effects of IL-4 on the immune system.

To the best of our knowledge, systemic IL-4 deficiency has not been reported to result in other immune-dysregulated disorders, including the spontaneous development of autoimmune symptoms, except that it makes the hosts vulnerable to parasitic infection (49). It may be argued that IL-4 is critical for maintaining Treg-mediated peripheral tolerance *in vivo*. In steady state, other cytokines, especially γ-chain cytokines, may play compensatory roles in an IL-4-deficient host. It has been demonstrated that Tregs can utilize other γ-chain cytokines than IL-2, including IL-4, IL-7, IL-15, and IL-21, to maintain their survival *in vitro* (38); we also found that the development of the Treg population was not affected in the IL-4-deficient host (Figures 1B,C). Nevertheless, we found Tregs from IL-4-deficient hosts demonstrated inferior suppressive capabilities for both antigen-specific and non-specific T cell activation, which strengthened the view that IL-4 is important for Treg-mediated immune suppression. Moreover, because lack of IL-4R mediated signaling does not make Tresps resistant to Treg-mediated immune suppression *in vitro* (36), IL-4 KO Tresp resistance to Treg-mediated suppression is unlikely the reason for the deteriorated suppression. Also, supplementation of exogenous IL-4 also rescued some suppressive capabilities of WT Tregs against IL-4 KO Tresp activation in the crisscross suppression assay. Therefore, the lack of IL-4 contributed to the incompetency of Tregs.

The beneficial effects of IL-4 on Tregs may act through STAT6-independent mechanisms (24). Granzyme B expression could be induced in human Tregs by treating cells with αCD3 plus αCD28 monoclonal antibodies with exogenous IL-2; the PI3K–mTOR signaling pathway might also be involved in this process (50). In murine CD4^+^ or CD8^+^ T cells, it has been suggested that IL-2 can induce granzyme B expression without TCR engagement (51). To date, the role of IL-4 on tuning granzyme expression has been demonstrated only on CD8^+^ T cells. Genetically engineered IL-4-secreting tumors enhanced both granzyme A and granzyme B expression in CD8^+^ T cells *in vivo* (52). In our study, we found increased granzyme A and granzyme B expression in Tregs when IL-4 was present in their surrounding environment. Future experiments will focus on identifying the underlying pathways that contribute to IL-4 mediated expression of granzymes in Tregs.

To conclude, our results demonstrated an extensive understanding of the contribution of IL-4 to Tregs and immune suppression. A physiologically relevant concentration of IL-4 can facilitate Treg-mediated immune suppression, perhaps through enhancing the expression of granzymes. Such findings benefit the design and applications of targeting STAT6 or IL-4-based therapeutic strategies for allergic and/or autoimmune diseases.

## Materials and Methods

### Mice

Mice at 6–8 weeks of age were utilized for experiments. IL-4 KO mice were a kind gift from Dr. John T. Kung (Academia Sinica, Taiwan) and OT2 mice were provided by Dr. Shih-Jen Liu (National Health Research Institutes, Taiwan). IL-4 KO and OT2 mice were bred and maintained in a specific pathogen-free barrier in the animal facility at Chang Gung University. Age- and gender-matched C57BL/6 mice were purchased from National Laboratory Animal Center (Taiwan) and used as wild-type controls for all experiments. Guidelines and regulations specified by the Institutional Animal Care and Use Committee of Chang Gung University (Taiwan) were strictly followed in handling the animals. The animal care, usage, and experimental protocols in this study were approved by the Institutional Animal Care and Use Committee of Chang Gung University.

### Medium and Culture Reagents

Complete medium for cell culture included αMEM (Thermo Fisher Scientific) supplemented with 10% heat-inactivated FBS (Merck), 55 nM β-mercaptoethanol, 100 U/mL penicillin, and 100 µg/mL streptomycin (Thermo Fisher Scientific). OT2 (OVA_323–339_ ISQAVHAAHAEINEAGR) peptide was obtained from Peptide Synthesis Core Facility of Academia Sinica and kept in a −80°C freezer until use.

### OVA Sensitization

The OVA sensitization protocol was modified from Yang et al. (53) Mice were intraperitoneally injected with a single shot of OVA (Merck) emulsion for three consecutive days (designated days 1–3) and then rested from days 4 to 12. A booster was given on day 13, and the mice were sacrificed on day 18. For each injection, 50 µg of OVA was emulsified in 20 µL of Alum Injection (Thermo Fisher Scientific), and PBS was added to bring the volume to 200 µL.

### CD4 T Cell Enrichment

For the *in vitro* suppression assay, CD4 T cells were enriched from murine splenocytes by EasySep Mouse CD4^+^ T Cell Enrichment Kit (STEMCELL Technologies) according to the manufacturer’s instructions with minor modifications. In brief, RBC removed splenocytes were resuspended at 1 × 10^8^/mL in HBSS supplemented with 5% (v/v) of normal rat serum and 2% (v/v) of heat-inactivated FBS. A mixture of biotinylated antibodies against non-CD4^+^ cells was added to the cell suspension, and the resultant cells were incubated at room temperature for 10 min. Streptavidin-coated magnetic beads were introduced to the tube, and a strong magnetic field was applied to remove non-CD4^+^ cells.

### Flow Cytometry and Cell Sorting

The expression levels of cell surface markers and intracellular transcription factors were analyzed by FACSCalibur (BD Biosciences), and cell sorting was performed with a FACSAria cell sorter (BD Biosciences). Phenotypic analysis and cell sorting were conducted with the following fluorochrome-conjugated monoclonal antibodies against murine antigens: anti-CD4-PerCP (RM4-5, BD Biosciences) and anti-CD25-APC (PC61.5, eBioscience). For Foxp3 analysis, cells were fixed and permeabilized with the Foxp3/Transcription Factor Staining Buffer Set (eBioscience) following the manufacturer instructed protocol. Intracellular Foxp3 was labeled by anti-Foxp3-PE (FJK-16s, eBioscience). Data were processed and analyzed using CellQuest or FACSDiva software (BD biosciences). Tregs (CD4^+^CD25^+^) and Tresps (CD4^+^CD25^−^) were sorted from enriched CD4 T cells. The purities of the sorted cells utilized in this study were all over 95%.

### Suppression Assay

Sorted Tresps (2 × 10^5^) were mixed with 2× serial diluted Tregs starting from 1:1 to 1:1/8 in a 96-well round bottom cell culture plate (Corning) containing 1 × 10^5^ of irradiated total splenocytes as accessory cells. Mixed cells were stimulated by 2.5 µg/mL of anti-CD3 (145-2C11, BioLegend) and 2.5 µg/mL of anti-CD28 (38.11, BioLegend) monoclonal antibodies in complete medium. OT2 Tresps were stimulated by 10 µg/mL OT2 peptide. In some experiments, 10 µg/mL anti-IL-4 antibody (11B11, BioLegend) or isotype control was added to neutralize IL-4. For IL-4 supplementary experiments, exogenous IL-4 (PeproTech) was introduced into the culture at the indicated concentrations. Cells were incubated at 37°C in a humidified cell incubator supplemented with 5% CO_2_. Cell proliferation was determined by ^3^H-thymidine incorporation assay.

### Determination of Gene Expression

Total RNA of cells was extracted by TRIzol Reagent (Thermo Fisher Scientific) according to the manufacturer’s instructions. Possible remaining genomic DNA was removed and cDNA was obtained by RT^2^ First Strand Kit (Qiagen). Real-time PCR was performed with RT^2^ SYBR Green qPCR Mastermixes (Qiagen). The PCR reactions and fluorescent signals were performed and analyzed with the CFX Connect Real-Time PCR Detection System (Bio-Rad). The primer pairs were as follows:

Gapdh forward, 5′-ACCCAGAAGACTGTGGATGG-3′
Gapdh reverse, 5′-ACACATTGGGGGTAGGAACA-3′
Gzma forward, 5′-GGAGAGCCACGATGAGGAAC-3′
Gzma reverse, 5′-AACAACCGTGTCTCCTCCAA-3′
Gzmb forward, 5′-ACAACACTCTTGACGCTGGG-3′
Gzmb reverse, 5′-CGAGAGTGGGGCTTGACTTC-3′.

### Statistical Analysis

The Student’s paired *t*-test, Student’s unpaired *t*-test, and one-way ANOVA with the *post hoc* test were performed by the statistical function in GraphPad Prism software to determine statistical significance. Values of *p* < 0.05 were considered statistically significant and indicated by asterisks on the figures.

## Ethics Statement

The study was carried out in accordance with the recommendations of the guidelines and regulations specified by the Institutional Animal Care and Use Committee of Chang Gung University (Taiwan). The animal care, usage, and experimental protocols in this study were approved by the Institutional Animal Care and Use Committee of Chang Gung University.

## Author Contributions

W-CY and C-RS conceived and designed the experiments; W-CY, Y-SH, Y-YC, W-HH, and S-ML performed the experiments; W-CY, Y-YC, and C-RS analyzed the data; Y-SH, C-LL, C-NS, and C-RS contributed to the protocol, reagents, materials, and analysis tools; and W-CY, C-LL, and C-RS wrote the manuscript. Y-SH, Y-YC, and C-LL contributed equally to this work.

## Conflict of Interest Statement

The authors declare that the research was conducted in the absence of any commercial or financial relationships that could be construed as a potential conflict of interest.
