# Supplementary material for: Interleukin-4 Supports the Suppressive Immune Responses Elicited by Regulatory T Cells
Source: Front Immunol. 2017 Nov 14;8:1508. doi: 10.3389/fimmu.2017.01508 (PMC5694475; doi:10.3389/fimmu.2017.01508)
Supplement: Supplementary file 1 [file data_sheet_1.pdf]

Supplementary Material

Interleukin-4 supports the suppressive immune responses elicited by regulatory T cells

Wei-Cheng Yang, Yih-Shiou Hwang, Ying-Yu Chen, Chao-Lin Liu, Chia-Ning Shen, Wei-Hsin Hong, Sheng-Min Lo, Chia-Rui Shen*

*** Correspondence:** Chia-Rui Shen: crshen@mail.cgu.edu.tw

Supplementary figure

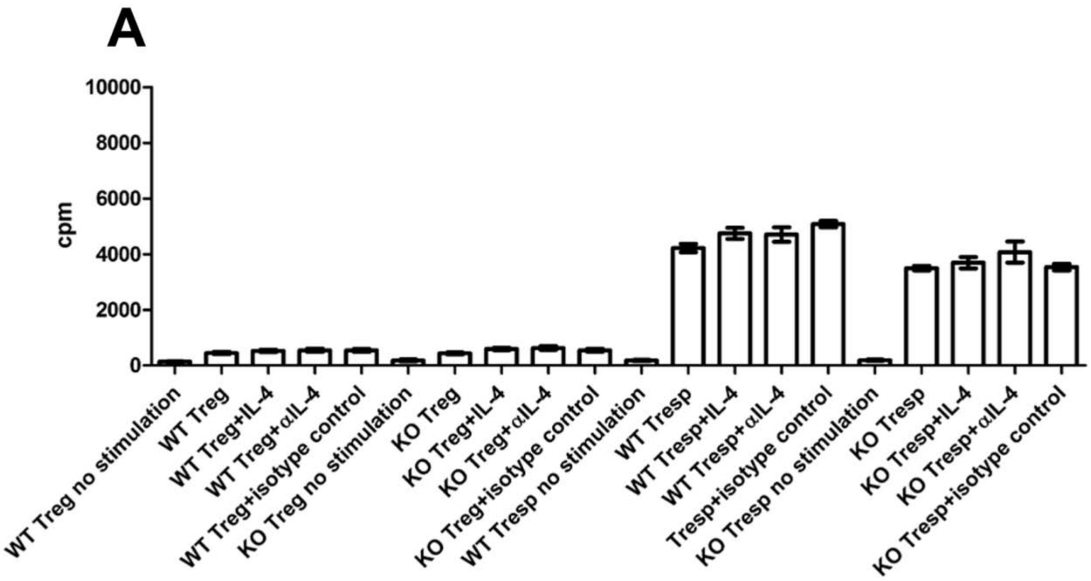

Supplement Figure 1. Tregs and Tresps from WT and IL-4 KO mice demonstrated no significant differences in their proliferation profiles when they were stimulated alone under different experimental circumstances.

(**A**) 2×10^5 of Tregs or Tresps from WT or IL-4 KO were mixed with 1×10^5 of irradiated total splenocytes as accessory cells and then activated by anti-CD3 plus anti-CD28 monoclonal antibodies for 3 days. The cell proliferation was determined by ^{3}H-thymidine incorporation assay. Data are representative of three independent experiments.

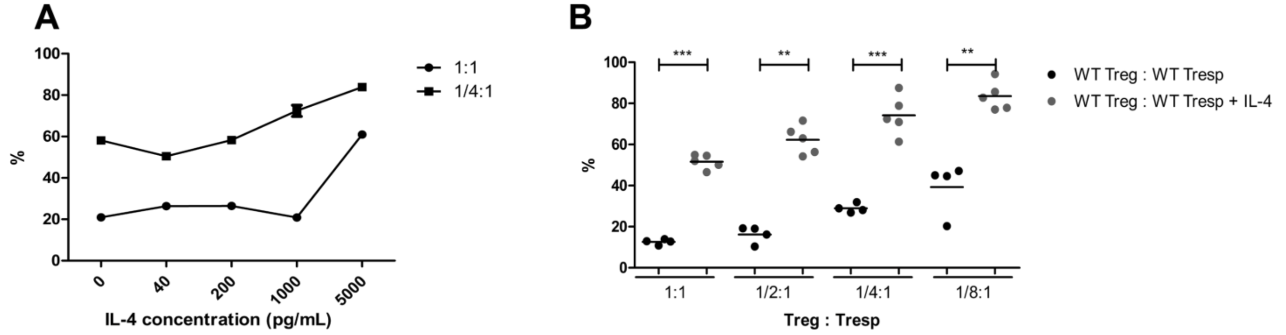

Supplement Figure 2. High dose IL-4 supplement did not benefit IL-4 KO and WT Treg cells mediated immune suppression

(**A**) IL-4 KO Treg cells mediated immune suppression was deteriorated when IL-4 was supplied at higher concentrations. (**B**) Supplement 10 ng/mL of IL-4 abrogated WT Treg cells mediated immune suppression. Results are presented as the relative percentage of cell proliferation to corresponding Tresps only. (*, p<0.05, **, p<0.01, ***, p<0.001)
